# Dementia Caregiver Burden: a Research Update and Critical Analysis

**DOI:** 10.1007/s11920-017-0818-2

**Published:** 2017-08-10

**Authors:** Sheung-Tak Cheng

**Affiliations:** 1 Department of Health and Physical Education, The Education University of Hong Kong, 10 Lo Ping Road, Tai Po, NT Hong Kong; 2 Department of Clinical Psychology, Noriwch Medical School, University of East Anglia, Norfolk, NR4 7TJ UK

**Keywords:** Dementia caregiving, Burden, Depression, Neuropsychiatric symptoms, Behavioral and psychological symptoms of dementia, Functional impairments

## Abstract

**Purpose of Review:**

This article provides an updated review of the determinants of caregiver burden and depression, with a focus on care demands and especially the differential effects of various neuropsychiatric symptoms or symptom clusters. Moreover, studies on caregivers for frontotemporal and Lewy body dementias were referred to in order to identify differences and similarities with the mainstream literature based largely on Alzheimer caregivers.

**Recent Findings:**

As a group, neuropsychiatric symptoms are most predictive of caregiver burden and depression regardless of dementia diagnosis, but the effects appear to be driven primarily by disruptive behaviors (e.g., agitation, aggression, disinhibition), followed by delusions and mood disturbance. Disruptive behaviors are more disturbing partly because of the adverse impact on the emotional connection between the caregiver and the care-recipient and partly because they exacerbate difficulties in other domains (e.g., caring for activities of daily living). In behavioral variant frontotemporal dementia, not only are these disruptive behaviors more prominent but they are also more disturbing due to the care-recipient’s insensitivity to others’ feelings. In Lewy body dementia, visual hallucinations also appear to be distressing.

**Summary:**

The disturbing nature of disruptive behaviors cuts across dementia conditions, but the roles played by symptoms that are unique or particularly serious in a certain condition need to be explored further.

## Introduction

A looming public health crisis is the rising number of people with dementia, doubling every 20 years, due to global aging. Currently, there are approximately 47 million people with dementia in the world [1], the great majority of whom are cared for by family members in the community [2]. A meta-analysis found dementia family caregivers to be significantly more stressed than non-dementia caregivers and to suffer more serious depressive symptoms and physical problems [3]. The situation may be more serious in developing countries where formal services and benefits for patients and caregivers are lacking [1]. Dementia caregivers are at risk for cardiovascular diseases, especially hypertension, which is mediated by chronic inflammatory response and sympathetic overactivation [4–6]. Another meta-analysis found overall prevalence rates of 34 and 44%, respectively, of elevated depressive and anxiety symptoms [7]. Using a diagnostic instrument, a longitudinal study found incidence rates of 37 and 55% for major depressive and anxiety disorders in a 24-month interval for caregivers without the conditions at baseline; comorbidities were high as 60% of caregivers had either depressive or anxiety disorder. Moreover, having subthreshold symptoms at baseline was the strongest predictor of developing a depressive or anxiety disorder later [8•]. Taken together, it appears that the majority of dementia caregivers are sufficiently disturbed to be of concern to the mental health professions.

Caregivers who are heavily burdened may opt for institutionalization of the relative as a role exit [9, 10], which in fact is associated with *increased* feelings of burden and depression in some caregivers following placement [11], along with adjustment issues and even early mortality of the relatives with dementia [12]. In another US study, caregiver depressive symptoms were found to be the most consistent predictor of increases in healthcare costs over an averaged 2-year period, including costs from the use of over-the-counter drugs [13]. As we head into the “dementia tsunami,” the burden on the health and social care system will be escalated unless family caregivers are properly supported.

This article provides an update and a critical overview of the recent literature on caregiver burden and depression, with a focus on care demands and especially the differential effects of individual neuropsychiatric symptoms (NPS) or NPS clusters on caregiver outcomes. In terms of caregiving outcomes, burden and depression are the two most researched constructs, whereas other relevant outcomes, such as anxiety and pre-death or anticipatory grief, have received much less attention.

The selective focus on caregiver burden and depression reflects only the state of the literature in which factors that influence these outcomes are better known. Note also that while burden and depression are treated as major outcome variables in this literature, they are not on the same level in the stress process as burden is theoretically an intermediate outcome, mediating the relationship between care demands and depressive symptoms [14]. Moreover, by focusing on the effects of care demands from the care-recipient (CR), this review will not cover caregiver factors in terms of both vulnerabilities (e.g., dysfunctional thoughts, lacking leisure/pleasant activities) and strengths (e.g., self-efficacy, positive growth), as well as contextual factors such as the availability of social support.

## Care Demands and Caregiver Burden

Research studies have consistently found NPS to be most disturbing to family caregivers, in comparison with other care demands such as the CR’s functional impairments or memory/cognitive deficits [15–18], although there are exceptions [19–21]. A well-cited meta-analysis of 228 studies found overall correlational coefficients with caregiver burden and depression, respectively, to be 0.37 and 0.27 for NPS, 0.22 and 0.14 for functional impairment, and 0.18 and 0.16 for cognitive impairment [15]. In addition, four long-term longitudinal studies showed that NPS early in the course of dementia [22], as well as subsequent increases in NPS [23–25], were most predictive of increases in burden scores over time. NPS are more distressing because they are unpredictable, disruptive, difficult to manage, potentially embarrassing or abusive, and sleep depriving.

Another literature, one that compares caregivers for relatives with the behavioral variant of frontotemporal dementia (bvFTD; a condition characterized by early behavioral and personality changes [26]) with AD caregivers, also provides indirect evidence concerning the stressful nature of NPS. Not surprisingly, bvFTD caregivers report more serious NPS in their CRs as well as higher levels of burden and depressive symptoms [27–30], although the extent to which the higher burden and depressive symptoms could be attributed to differences in NPS was not clear as researchers did not include NPS as a covariate in the analysis. The range of NPS co-occurring, as well as their frequencies, at the early stage of bvFTD may exacerbate stress reactivity on the part of the caregiver. One study found that the correlation between NPS and burden, though statistically significant, was much lower in AD caregivers (*r* = 0.35) than in bvFTD caregivers (*r* = 0.70) [29].

Two studies on NPS and cortisol, a biomarker of stress and hypothalamic-pituitary-adrenal axis activity, found different results. Compared with noncaregivers, dementia caregivers were found to have elevated cortisol levels at the time of awakening, but similar levels throughout the day. However, the pattern for the caregivers was different depending on their relative’s NPS level. While caregivers reporting low NPS in their relatives had a blunted morning rise in their cortisol levels, those reporting high NPS showed a typical morning rise to 30 min post-awakening and they also showed a steeper decline of cortisol for the rest of the day [31]. Another study investigated variations in cortisol levels in the same caregiver from day to day (i.e., within-subjects design), instead of simply examining mean levels of the whole sample. Diurnal salivary cortisol samples were collected on 4 days over a 1-week period from 30 caregivers for relatives with mild cognitive impairment. Controlling for caregiver sleep disturbance and physical and depressive symptoms, the researchers found elevated cortisol levels throughout the day on days when NPS (especially mood disturbance) were present than on days without NPS [32, 33]. Although more research is needed to clarify the relationship between NPS and caregivers’ cortisol response, the preliminary data add further to the evidence concerning the stressful impact of NPS.

Although NPS, compared with functional impairments, tend to emerge as the stronger predictor of caregiver burden and depression, such a finding may be an artifact of the characteristics of the CRs enrolled in the study. For a number of reasons, participants in the majority of research studies thus far tend to be caregiver-CR dyads who are (near) midway in the course of Alzheimer’s disease (AD), when NPS dominate the clinical picture. For example, the mean care duration at study entry has been reported to be around 3–4 years in most studies [16, 23, 34]. This is usually the time when NPS become prominent in the course of AD, although the timing at which different symptoms peak varies and certain symptoms are not as persistent as others [35, 36]. Yet, in advanced dementia, when bodily systems are shutting down, NPS will eventually decrease and functional dependencies will dominate the attention of caregivers [37]. Although caregivers of such late-stage patients are rarely represented in research studies, there is at least some evidence that burden increases with dementia severity [28, 38, 39] and a major reason for this is the increasing personal involvement of the caregiver in terms of the number of daily care hours due to impairments in basic and instrumental activities of daily living (ADLs) [38]. Note also that caregivers who remain in studies and are still caring for someone with advanced dementia are probably those who have adapted more successfully (as those who feel overwhelmed may have placed their relatives in institutions) and so existing studies may have underestimated the levels of caregiver burden in advanced dementia.

A large survey of caregivers in five European countries including Germany, France, Poland, Scotland, and Spain was illustrative. Anonymous questionnaires were distributed via member organizations of Alzheimer Europe in these countries and 1181 caregivers responded; responses were based purely on caregivers’ self-report. Only 72% of the caregivers could provide information on the stage of the dementing illness of their CRs; among these, about 11, 53, and 36% were taking care of someone in the mild, moderate, and severe stage, respectively. There was a more or less dose-dependent relationship between dementia severity and care hours, with 20% (mild), 39% (moderate), and 50% (severe) of the caregivers providing >10 h of care per day. Another one third of the CRs with mild dementia, and one quarter of those with moderate or severe dementia, received 4–10 h of daily care from their caregivers. Overall, one third of the caregivers (disregarding CR dementia stage) provided >14 h of care on a daily basis. As for the most problematic areas (multiple options allowed), ADL impairments (e.g., bathing, toileting) were named by 68% of the caregivers, followed by behavior problems (50%), cognitive impairments (45%), and communication problems (36%) [40]. Thus, in a sample where there were more severe cases, ADL impairment may emerge as a more prominent concern for caregivers. Another US study corroborated these descriptive data by showing that functional dependency was more strongly correlated with the number of care hours than NPS, and was the only factor independently associated with missing more hours at work for those who were employed [18]. Note that the length of time providing care on a day-to-day basis, an objective burden indicator, is one of the most consistent predictors of subjective burden and depression [15, 16, 41, 42].

Furthermore, research on caregivers for relatives having dementia with Lewy bodies (DLB), a condition characterized by more serious ADL impairments [43], also shows such impairments, but not Parkinsonian motor signs, to be a significant factor in caregiver burden [44]. For instance, one study showed that ADL impairment was a stronger predictor of role strain than NPS [45].

Two comments need to be made. In dementia, the burden of providing care for ADLs is often exacerbated by NPS. For example, the CR may display verbal or physical aggression when the caregiver attempts to bath, to dress, or to feed him or her. Fauth and colleagues found that NPS that occurred in the context of ADL assistance (i.e., resistiveness to care) largely accounted for the associations between ADL impairments and measures of burden (role captivity and overload) and depressive symptoms, so that these associations became nonsignificant after controlling for the frequency of resisting care [46•]. There is also a possibility that certain NPS (e.g., hyperorality) are reported as an ADL issue (e.g., feeding) by caregivers. Apart from sample characteristics (i.e., most CRs in moderate stage) noted above, the contribution of NPS to ADL problems may suggest why, although ADL assistance accounts for the majority of caregiving load in terms of the time spent and physical exertion, ADL impairment is frequently the less important predictor of caregiver burden and depression, compared with NPS. It also suggests that resistiveness to care may partially mediate the relationship between NPS and caregiver outcomes, a hypothesis that needs to be tested in future research. The same may be said about the relationship between cognitive impairment and caregiver outcomes as confusion often underlies resistiveness to care.

Another issue concerns whether functional dependencies and NPS impact on different burden dimensions. A well-designed, though cross-sectional, study investigated the contributions of various primary stressors (e.g., behavioral problems [NPS], cognitive impairments, and physical dependencies) and contextual factors (e.g., whether living together, ethnicity, relationship to CR, whether employed) to burden and depressive symptoms. It is noteworthy that while existing studies have typically focused on a global measure of burden (e.g., the Zarit Burden Interview [47]), the researchers included four different dimensions of burden, namely relationship strain, social isolation strain, emotional strain, and physical health strain, thus enabling an examination of the associations between different stressors and different aspects of burden. Judging from the regression coefficients, the order of the influence of the different primary stressors of interest was (from high to low unless otherwise specified) as follows: *depressive symptoms*—behavioral and physical; *relationship strain*—behavioral; *social isolation*—physical, cognitive, and behavioral; *emotional strain*—behavioral, cognitive, and physical; *physical strain*—behavioral and physical being equally predictive (only stressors that were significant are listed here) [48•]. The regression coefficients of the individual predictors had not been subject to formal tests to show that they were significantly different from each other. Nevertheless, the pattern suggests that when researchers examine the *overall* burden score or depressive symptom score as a dependent variable, NPS would generally be found to be the most powerful predictor, followed by physical/functional dependency, and lastly cognitive impairment. This was, to my knowledge, the first time that different stressors and contextual variables were analyzed in such a systematic fashion against various outcome variables. It should come as no surprise that caring for functional impairment was most strongly associated with social isolation strain, although NPS, especially those found to be embarrassing, could also restrict social activity. Also worthy of noting was that increased relationship strain was associated with higher NPS scores, as behavior problems often create tension between the caregiver and the CR. Thus, instead of simply asking which type of problem is more burdensome to caregivers, it may be more productive to analyze the ways by which the care demands impact on the different dimensions of burden. Doing this would necessitate the assessment of these dimensions separately.

## Individual Neuropsychiatric Symptoms or Symptom Clusters

Although collectively NPS present the greatest difficulty for caregivers, a question remains as to whether certain symptoms are more challenging than others and are more useful indicators of the caregiver’s need for assistance or intervention. Note that because depression is one of the NPS as well as the main focus of caregiver outcome here, terms such as “dysphoria/depression” or “mood disturbance” will be used to refer to the former, reserving “depressive symptoms” to the latter, to improve readability. This question may be explored by asking caregivers the degree to which they are bothered by the different symptoms or by analyzing the degree to which the symptoms or symptom clusters differentially predict burden and related measures. For the former approach, it is interesting that two recent studies, one in the USA and the other in Taiwan, converged to suggest that delusions, agitation/aggression, and irritability were the most distressing to caregivers, while anxiety and dysphoria/depression also tended to cause distress [49, 50].

These findings were consistent with those in the original validation study of the Neuropsychiatry Inventory-Distress scale (NPI-Distress) [51], suggesting a good degree of consistency across time and culture. For instance, using the Revised Memory and Behavior Problems Checklist [52] and comparing the average reaction ratings for the three subscales (i.e., disruptive behaviors, mood disturbance, and memory-related behaviors), Fauth and Gibbons found disruptive behaviors to be most disturbing to caregivers, followed by mood disturbance, and lastly memory-related behaviors (psychotic symptoms not measured by this checklist) [49]. These findings should not be surprising considering the irritating and embarrassing nature of certain behaviors (e.g., swearing, sexual disinhibition, physical agitation, stereotypical behaviors) and the common delusions (e.g., persecution, infidelity).

Another approach researchers have taken would be to conduct multiple regression analysis using different NPS as individual predictors of caregiver burden and depressive symptoms. Controlling for burden and instrumental ADLs, a Japanese study found only delusions to be associated with elevated depressive symptoms in the caregivers [20]. However, Dauphinot and colleagues found the presence of apathy, agitation/aggression, aberrant motor behavior, appetite changes, and irritability to be significantly and independently associated with burden in 548 French caregivers, after controlling for global cognitive performance, functional impairment, and use of antidepressants [19].

Such analysis of individual symptoms is not always feasible due to the co-occurrence of certain symptoms in some individuals, making it difficult to determine the unique effect of each symptom. Several researchers have elected to examine the clustering of symptoms and their effects [53–56] in order to minimize statistical redundancy. On the basis of three clusters of NPI symptoms established via confirmatory factor analysis [57], Cheng and colleagues found that the disruptive behavior cluster (i.e., agitation/aggression, irritability, disinhibition, aberrant motor behavior) was the strongest and most consistent predictor of measures of burden and depressive symptoms in AD caregivers, followed by the mood cluster (e.g., anxiety, dysphoria/depression), while the psychosis cluster was unrelated to the outcomes [16]. But whether the effect of delusions was attenuated by the simultaneous inclusion of hallucinations within the psychosis cluster was not clear.

A team of researchers grouped the symptoms conceptually, but they did it inconsistently. In a cohort of caregivers followed for an average of 4.5 years, they initially placed disruptive behaviors into two different categories (e.g., grouping aggression together with paranoid and abandonment delusions, but placing agitation, wandering, and verbal outbursts together with sundowning in another group). Using this grouping method, they found that only mood disturbance (depression), when present, was significantly correlated with caregiver burden and depressive symptoms at baseline, after controlling for CR functional impairment and whether the caregiver was a spouse [58]. However, when examining the longitudinal outcomes, they regrouped agitation, verbal outburst and physical aggression into a new category and found that this symptom cluster, when persisted during the first year of study, was the only one predicting subsequent depressive symptoms in the caregiver [59].

These longitudinal findings were replicated by another large-scaled, multinational study of European and North American caregivers for persons with Parkinson’s disease dementia (PDD; *N* = 537). The researchers grouped the participants according to their CRs’ NPS profiles using cluster analysis and analyzed the relationships between NPS profiles and overall NPI-Distress scores. It was found that those caring for CRs with more irritability and agitation (the agitation profile) and those whose CRs had more visual hallucinations and delusions reported highest distress, over those with CRs having the apathy or the mood profile. Agitation and psychosis also got worse with Parkinson’s disease progression [60]. That these two NPS profiles were associated with more burdened caregivers was replicated in a small sample of Korean PDD caregivers [61]. Thus, in addition to disruptive behaviors and delusions, visual hallucination, a symptom more serious and early in the course of Lewy body dementia including PDD [43], is also very stressful for these caregivers.

As mentioned before, a pathway by which NPS affect caregiver well-being is the adverse impact on the emotional connection with the CR, which may be more pronounced in CRs with more serious disruptive behaviors. The damage to the mutual relationship was found to be one of the factors underlying the higher burden among caregivers for relatives with behavioral variant frontotemporal dementia (bvFTD), a condition characterized by early and more serious behavioral and personality changes related to frontal systems (e.g., executive disinhibition and apathy) [26], than among caregivers for AD relatives [28]. The irritating nature of the disruptive behaviors is likely exacerbated by impairments in insight and empathy. An interesting proposition is that stress in bvFTD caregivers is related to the CR’s loss of empathy as well as lack of insight about the awkward and insensitive nature of their behaviors that affect the relationship with others including the caregiver [62–64]. It is noteworthy that Wong and Wallhagen added eight items, including loss of insight, on top of the 12 symptoms measured by the NPI, and found loss of insight to be very distressing to caregivers [65].

In general, people with bvFTD are reported to have more frequent or serious agitation, disinhibition, apathy, and eating abnormalities, and these are also the symptoms identified to be the more upsetting ones on the NPI-Distress scale by their caregivers [30, 65, 66]. Apathy and immobility may be more upsetting in bvFTD than in AD because in the former, they are manifested with intermittent outbursts of disinhibition and restlessness [67], and so apathy might not be independently associated with burden in FTD caregivers when the other NPS, especially disruptive behaviors, were controlled for [68].

Moreover, the rates of disruptive behaviors reported by Asian caregivers appear to be higher than those reported in Western studies [57, 69–71]. For instance, twice as many Singaporean caregivers as French caregivers recruited from clinical settings reported experiencing agitated and aggressive behaviors from their CRs [69, 70]. Such cross-cultural patterns may be due to the relative lack of services in Asian societies and the emphasis on harmony and order in these societies so that caregivers are less tolerant of these behaviors. The reasons behind need to be investigated in future research.

## Concluding Remarks and Future Research

Several observations can be highlighted from this review. First, although NPS tend to present more challenges than cognitive and functional impairments, there is a need to look beyond the global score to examine the relative contributions of different NPS to caregiver burden and depressive symptoms. The current evidence suggests that various disruptive behaviors are most upsetting partly because of the effect on the caregiver’s relationship with the CR and partly because of exacerbating difficulties in providing ADL assistance and in other domains. The stressful nature of these symptoms appears to cut across dementia conditions, which may be used to guide the prioritization of caregivers, if necessary, for interventions. These behaviors may also cause distress partly by engendering a sense of loss of control, which may explain why they are more problematic for Asian caregivers, but no data are available to speak to this point yet.

In addition to disruptive behaviors, delusions in the CR also tend to be very distressing to the caregiver although it is not clear whether all kinds of delusion are equally distressing. For example, delusions of persecution and infidelity may create tension between the caregiver and the CR more so than misidentification. However, assembling enough CRs with different kinds of delusions to enable a comparative analysis may not be at all feasible. Compared with delusions and disruptive behaviors, dysphoria/depression and anxiety were less consistently identified as distressing to caregivers.

Second, while the literature is dominated by studies on the challenges faced by AD caregivers, more studies are needed to investigate the situations of non-AD caregivers. Due to space limitations, the present review is intended to highlight some key trends in selected disorders, focusing on findings that have been replicated. The findings to date suggest similar patterns in bvFTD and DLB/PDD caregivers in that disruptive behaviors in the CR are among the most problematic and disturbing symptoms. However, few studies have assessed the range of symptoms in these conditions comprehensively, not to mention the typically small samples that do not permit the simultaneous analysis of the different symptoms. What may be discerned from the current literature is that visual hallucinations, along with delusions, are quite disturbing to Lewy body dementia caregivers, but the effects of sleep disturbance and mental fluctuations need to be further researched [72]. Also, requiring further research is the role of apathy in bvFTD caregiver burden.

Third, just as researchers need to go beyond the global score aggregated from all NPS, they need to improve the measurement of the different dimensions of burden to enable more refined analysis of the effects of different care demands on caregiver burden. This is because different factors may impact on different burden dimensions, as in the case when caring for the CR’s ADLs may restrict the caregiver’s leisure and social activities more so than other care duties. Moreover, not all dimensions of burden (e.g., financial strain) are amenable to interventions and a multidimensional instrument may offer flexibility in the choice of dimensions suitable as outcome measures in specific contexts.

As the dementia tsunami approaches, a better understanding of the sources and indicators of caregiver stress can help researchers and practitioners identify the most appropriate and needy caregivers for different interventions. By using a broader set of criteria such as the types and levels of care demands, the kinds of impact on the caregiver’s life (i.e., aspects of burden), and/or the presence of elevated psychiatric symptoms, service providers will be in a more informed position to match intervention types to the needs of different caregivers. As was alluded to earlier, the present review should not be taken to mean that problems presented by the person with dementia are the only reasons that caregivers are stressed, as stress depends also on personal resources in adaptation to challenging situations [73, 74]. For example, a recent intervention adopts methods based on cognitive therapy to promote caregiver resilience and positive growth, resulting in decreased burden and depressive symptoms [75••, 76]. Due to the space limitation, however, the literature on protective factors was not covered here although these factors are equally important for determining caregivers who require assistance. When the conditions leading to negative mental health are better understood, caregivers at risk may be identified for interventions which can be used for preventive as well as remedial purposes.
